# Supplementary material for: SurfDiff: protein surface profiling for selective or broadly reactive epitope prioritisation in binder and immunogen design
Source: bioRxiv. 2025 Dec 23:2025.12.23.696170. Preprint. [Version 1] doi: 10.64898/2025.12.23.696170 (PMC12776457; doi:10.64898/2025.12.23.696170)
Supplement: Supplement 1 [file media-1.pdf]

SUPPLEMENTARY INFORMATION

SurfDiff: protein surface profiling for selective or broadly reactive epitope prioritisation in binder and immunogen design

Benedikt Jedlitzke[1], Oliver Wissett[1], Tung H. Nguyen**[1,2,3], Elodie Ghedin[2], and Pietro Sormanni[1,4,*]**

[1] Centre for Misfolding Diseases, Yusuf Hamied Department of Chemistry, University of Cambridge, CB2 1EW, Cambridge, UK
[2] Systems Genomics Section, Laboratory of Parasitic Diseases, National Institute of Allergy and Infectious Diseases (NIAID), National Institutes of Health (NIH), Bethesda, Maryland 20894, USA
[3] School of Medicine and Public Health, University of Wisconsin, Madison, Wisconsin 53705, USA
[4] Department of Chemical Engineering, Imperial College London, South Kensington Campus, London, SW7 2AZ, UK
*Correspondence to: pietro.sormanni@imperial.ac.uk

SUPPLEMENTARY FIGURES

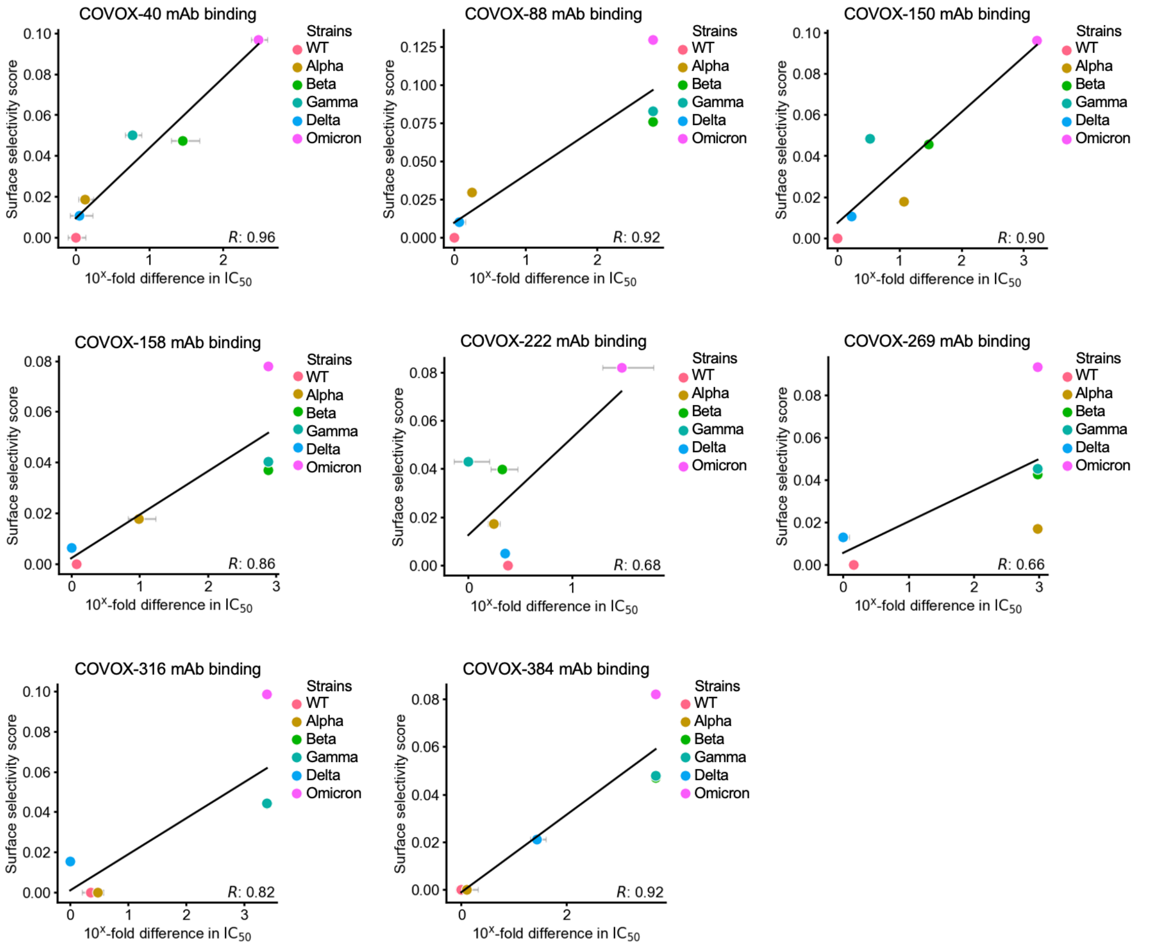

Figure S1: Correlation between surface selectivity scores and antibody inhibitory potency differences across SARS-CoV-2 variants for individual COVOX antibodies. Scatter plots show the relationship between SurfDiff surface selectivity scores and experimentally determined inhibitory potency differences (expressed as $\log_{10}(IC_{50}\_variant / IC_{50}\_WT)$) for each of the eight COVOX antibodies. Surface selectivity scores were computed over antibody epitopes defined from co-crystal structures using a 4 Å heavy atom distance cutoff. Each panel corresponds to a different antibody and presents the Pearson correlation coefficient (*R*) between surface selectivity and log-fold changes in neutralisation potency across SARS-CoV-2 variants. Variants that showed no neutralisation were assigned an IC_{50} of 20 µg/mL, which is twice the reported detection limit.

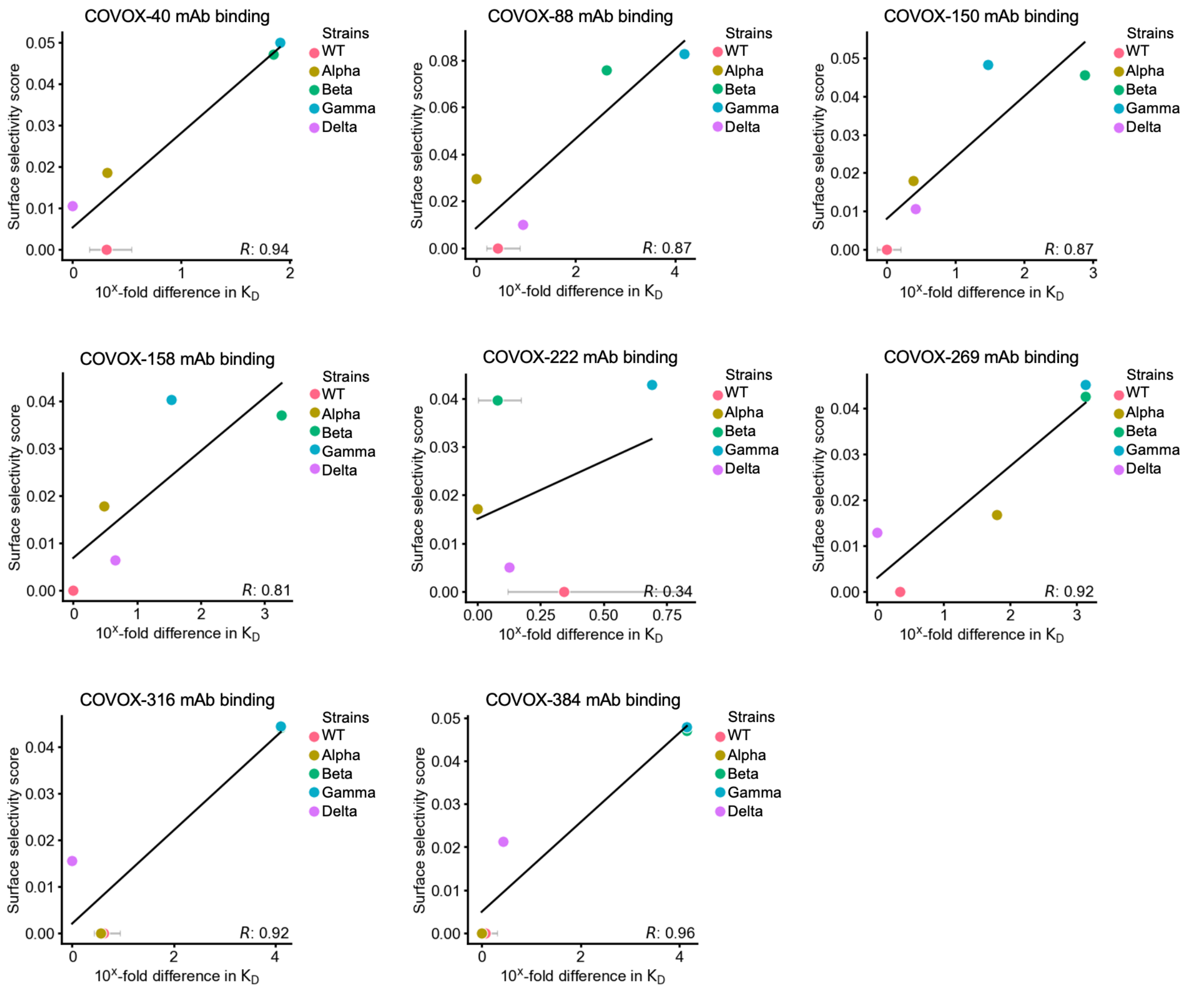

Figure S2: Correlation between surface selectivity scores and antibody binding affinity differences across SARS-CoV-2 variants. Scatter plots showing the relationship between SurfDiff surface selectivity scores and experimentally determined binding affinity differences ($\log_{10}(K_D\_variant / K_D\_WT)$) for each of the eight COVOX antibodies. Pearson correlation coefficients (*R*) are reported for each antibody. This panel is analogous to Supplementary Figure S1, but uses K_D values instead of IC_{50}. Variants that showed no binding affinity were assigned a K_D of 10 µM, which is one magnitude higher than the reported detection limit.

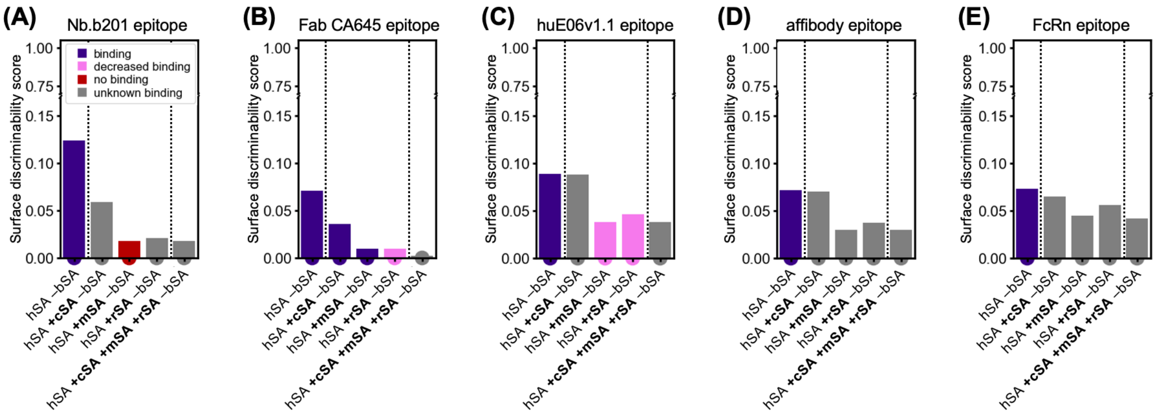

Figure S3: Surface discriminability score analyses for multiple human serum albumin (hSA) binders. Bar plots of surface discriminability scores for the epitopes of six experimentally characterised binders: nanobody Nb.b201, which was isolated with yeast display from a synthetic library (known to bind hSA and not mSA, while bSA was in the selection buffer so it's unlikely to bind that too). Fab CA645, shark VNAR huE06v1.1, an affibody (Affibody® ABD/GA), and the human neonatal Fc receptor (FcRn). Each panel displays the discriminability score at the respective epitope across hSA and its orthologues, enabling comparison of predicted cross-reactivity with reported experimental binding data.

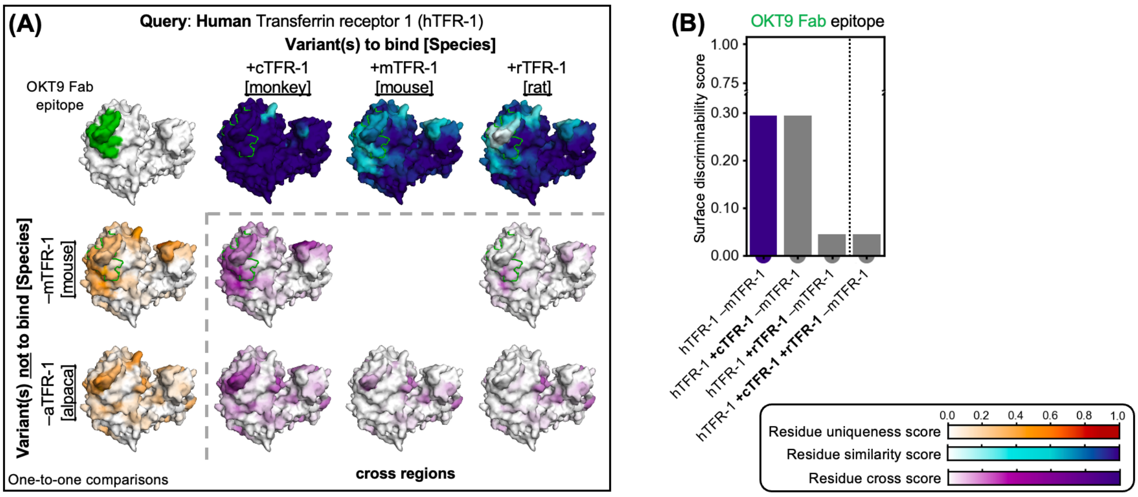

Figure S4: Identification of potential cross-reactive epitopes across transferrin receptor 1 (TFR-1) orthologues using SurfDiff. (A) Residue similarity and uniqueness scores mapped onto the surface of human TFR-1 (AlphaFold model, UniProt P02786) in comparison with orthologues from mouse, rat, alpaca, and cynomolgus monkey. Left: residue uniqueness scores relative to potential immunisation species (mouse, alpaca) highlight regions likely to elicit an immune response. Top: residue similarity scores relative to potential preclinical species (monkey, mouse, rat) highlight regions that may support cross-reactivity. Middle: surface discriminability scores integrate both metrics, identifying epitope regions that are distinct from immunisation hosts yet conserved across preclinical species. The OKT9 Fab epitope (AlphaFold3 model with docking-based orientation[1]) is shown in green.**(B)** Surface discriminability scores at the OKT9 epitope across variant(s) to bind. High scores for human are consistent with the known OKT9 binding profile, while low scores for mouse align with its lack of binding, reflecting development through mouse immunisation. A high score for monkey suggests potential cross-reactivity, although this has not been experimentally validated. In contrast, the low score for rat indicates limited predicted cross-reactivity, though experimental data are not available.

SUPPLEMENTARY TABLES

Cluster ID	Seed residue	Residues	Cluster size	Surface discriminability score [-]	Epitope SASA [Å^2]
1	324	[324, 325]	2	0.37	236.0
2	528	[528, 529]	2	0.28	209.0
3	85	[85, 88, 87]	3	0.22	241.0
4	340	[340, 344]	2	0.21	97.0
5	141	[141]	1	0.19	92.0
6	585	[585]	1	0.18	25.0
7	96	[96]	1	0.15	56.0

Table S1: Top-ranked epitope clusters on the surface of human serum albumin (hSA) with the highest surface discriminability scores for hSA +cSA +mSA +rSA –bSA –aSA. Binding variants considered: hSA, cynomolgus monkey (cSA), mouse (mSA), and rat (rSA). Non-binding variants considered: alpaca serum albumin (aSA) and bovine serum albumin (bSA). Cluster information includes the cluster ID, the seed residue (UniProt residue numbering), all residues within the cluster (UniProt residue numbering), total number of residues, mean surface discriminability score, and solvent-accessible surface area (SASA). Query PDB: modelled with AF3 and signal peptide (residue 1-24) removed, UniProt ID: P02768).

Cluster ID	Seed residue	Residues	Cluster size	Surface discriminability score [-]	Epitope SASA [Å^2]
1	103	[103]	1	0.42	25.0
2	30	[30, 31, 32]	3	0.33	358.0
3	31	[31, 30, 32, 135]	4	0.28	404.0
4	68	[68]	1	0.27	35.0
5	32	[32, 135, 31, 34, 30]	5	0.26	468.0
6	133	[32, 34, 36, 37, 38, 55, 133, 135]	8	0.20	381.0
7	37	[37, 36, 38, 53, 55]	5	0.20	200.0
8	38	[38, 36, 37, 53]	4	0.20	174.0
9	53	[53, 38, 55, 37]	4	0.20	134.0

Table S2: Top-ranked epitope clusters on the surface of human PD-1 (hPD-1) with the highest surface discriminability scores for hPD-1 +cPD-1 +rPD-1 –mPD-1. Binding variants considered: hPD-1, cynomolgus monkey (cPD-1) and rat (rPD-1). Non-binding variants considered: mouse PD-1 (mPD-1). Cluster information includes the cluster ID, the seed residue (UniProt residue numbering), all residues within the cluster (UniProt residue numbering), total number of residues, mean surface discriminability score, and solvent-accessible surface area (SASA). Query PDB: 8gy5.

Cluster ID	Seed residue	Residues	Cluster size	Surface discriminability score [-]	Epitope SASA [Å^2]
1	127	[127, 132]	2	0.24	78.0
2	114	[114, 116]	2	0.14	221.0

Table S3: Top-ranked epitope clusters on the surface of human PD-1 (hPD-1) with the highest surface discriminability scores. Binding variants considered: hPD-1, cynomolgus monkey (cPD-1), mouse (mPD-1), and rat (rPD-1). Non-binding variants considered: alpaca PD-1 (aPD-1). Cluster information includes the cluster ID, the seed residue (UniProt residue numbering), all residues within the cluster (UniProt residue numbering), total number of residues, mean surface discriminability score, and solvent-accessible surface area (SASA). Query PDB: 8gy5.

**A**	0.00																				
**R**	0.67	0.00																			
**N**	0.78	0.56	0.00																		
**D**	0.78	0.78	0.44	0.00																	
**C**	0.56	0.83	0.78	0.78	0.00																
**Q**	0.67	0.44	0.56	0.56	0.83	0.00															
**E**	0.67	0.56	0.56	0.33	0.89	0.33	0.00														
**G**	0.56	0.78	0.56	0.67	0.78	0.78	0.78	0.00													
**H**	0.78	0.56	0.50	0.61	0.78	0.56	0.56	0.72	0.00												
**I**	0.67	0.94	0.89	0.89	0.67	0.94	0.94	1.00	0.89	0.00											
**L**	0.67	0.78	0.89	1.00	0.67	0.78	0.94	1.00	0.89	0.28	0.00										
**K**	0.67	0.33	0.56	0.67	0.83	0.44	0.44	0.78	0.67	0.94	0.78	0.00									
**M**	0.67	0.67	0.78	0.89	0.67	0.56	0.78	0.89	0.72	0.44	0.33	0.67	0.00								
**F**	0.78	0.89	0.83	0.83	0.72	0.89	0.89	0.83	0.61	0.56	0.56	0.89	0.56	0.00							
**P**	0.67	0.72	0.72	0.67	0.78	0.67	0.67	0.72	0.72	0.89	0.89	0.67	0.72	0.89	0.00						
**S**	0.44	0.67	0.44	0.56	0.67	0.56	0.56	0.56	0.67	0.83	0.83	0.56	0.67	0.78	0.67	0.00					
**T**	0.56	0.67	0.56	0.67	0.67	0.67	0.67	0.78	0.72	0.67	0.67	0.67	0.67	0.78	0.67	0.44	0.00				
**W**	0.83	0.78	0.83	0.83	0.67	0.72	0.78	0.72	0.67	0.83	0.72	0.78	0.61	0.50	0.83	0.83	0.72	0.00			
**Y**	0.78	0.72	0.72	0.83	0.72	0.67	0.72	0.83	0.39	0.67	0.67	0.72	0.67	0.28	0.78	0.78	0.72	0.44	0.00		
**V**	0.56	0.94	0.89	0.89	0.67	0.78	0.78	0.89	0.89	0.11	0.44	0.78	0.44	0.67	0.78	0.83	0.56	0.83	0.67	0.00	
**-**	1.00	1.00	1.00	1.00	1.00	1.00	1.00	1.00	1.00	1.00	1.00	1.00	1.00	1.00	1.00	1.00	1.00	1.00	1.00	1.00	
**Aa**	**A**	**R**	**N**	**D**	**C**	**Q**	**W**	**G**	**H**	**I**	**L**	**K**	**M**	**F**	**P**	**S**	**T**	**W**	**Y**	**V**	**-**

Table S4. Residue Chemical Distance matrix used in SurfDiff calculations. This matrix was derived from a modified BLOSUM62 substitution matrix reformulated to represent physicochemical distances rather than evolutionary background frequencies. Diagonal values (self-matches) were set to zero to isolate identical substitutions. The resulting scores were min–max normalised to span the range from 0 to 1, providing a scale interpretable as physicochemical dissimilarity for each residue (Aa). Residues classified as different (–) were assigned the maximum score of 1.00.

	**PDB-ID / AF3 (UniProt-ID)**	**Binding complex with**
**SARS-CoV-2 (RBD) strains**		
WT [Victoria]	7neh / 7z0x / 7nd3 / 7bel / 7bei / 7qny / 7nx6 / 7beh / 7bep	COVOX-40 (7nd3), -88 (7bel), -150 (7bei), -158 (7qny), -222 (7nx6), -269 (7neh), -316 (7beh), -384 (7peb)
Alpha [B.1.1.7]	7ek0	
Beta [B.1.351]	7ps7	
Gamma [P.1]	7nxc	
Delta [B.1.617.2]	7x7d	
Omicron [B.1.1.529]	7qnw	
**Serotonin receptor subtypes**		
5-HT1A	7e2y / 7e2z	serotonin (7e2y)
5-HT1B	6g79	
5-HT1D	7e32	
5-HT1E	7e33	
5-HT1F	7exd	
5-HT2A	7ran / 8uwl	lisuride (8uwl)
5-HT2B	7srr	
5-HT2C	8dpf	
5-HT4	7xt8	

5-HT5A	7um7	
5-HT6	8jlz	
5-HT7	7xtc	5-carboxamido-tryptamine
**Dopamine receptor subtypes**		
D1	7jvp	
D2	7jvr	
D3	7cmv	
D4	8iru	
D5	8irv	
**Adrenergic receptor subtypes**		
Aa1A	8thk	
Aa1B	7b6w	
Aa1D	AF3 (P25100)	
Aa2A	7ej8	
Aa2B	6k41	
Aa2C	6kuw	
Ab1	7bu6	
Ab2	4lde	
Ab3	9ijd	
**Vasopressin / Oxytocin receptor subtypes**		
V1A	AF3 (P37288)	
V1B	AF3 (P47901)	
V2	7dw9	vasopressin
OT	7qvm	oxytocin
**Somatostatin receptor subtypes**		
SSTR1	8xio	
SSTR2	7t10	somatostatin-14
SSTR3	8xir	
SSTR4	7xms	
SSTR5	8x8n	
**Dengue virus (DIII envelope protein) strains**		
DENV-1 [FGA89]	3uzq / 4l5f	mAb 4E11 (3uzq)
DENV-2 [1409]	8y3k	
DENV-3 [PaH881]	8jn4	
DENV-4 [63632]	3uyp	
**HIV (gp120 envelope protein) strains**		
HIV-1 [Q23.17]	4ydi	
HIV-1 [KER_2018_11]	4lss	
HIV-1 [Q842.d12]	4xmp	
HIV-1 [93TH057]	5te6 / 4xvt	mAb N6 (5te6)
HIV-1 [YU2]	3tgg	
HIV-1 [ZM176.66]	4lst	
HIV-1 [C1086]	4lsv	
HIV-1 [DU172.17]	5te7	
HIV-1 [X2088]	5te4	
HIV-2	5cay	
**AngiotensinII receptor subtypes**		
ATR1	6os2	
ATR2	6jod / 5xjm	AngII (6jod) / 4A03 Fab (6jod)
**Transforming growth factor-beta cytokine isoforms**		

TGF-β1	4kv5	fresolimumab
TGF-β2	4kxz	fresolimumab
TGF-β3	3eo1	fresolimumab
**Serum Albumin species orthologs**		
Human [homo sapiens]	AF3 (P02768) / 8oi2 / 5vnw / 5fuo / 1tf0 / 4n0f	NbAlb1 (8oi2), Nb.b201 (5vnw), Fab CA645 (5fuo), affibody (1tf0), FcRn (4n0f)
Monkey [cynomolgus monkey]	AF3 (A2V9Z4)	
Mouse [mus musculus]	AF3 (P07724)	
Rat [rattus norvegicus]	AF3 (P02770)	
Bovine [bos taurus]	AF3 (P02769)	
Alpaca [lama pacos]	AF3 (A0A6I9IHM8)	
Shark	4hgm	huE06v1.1
**PD-1 species orthologs**		
Human [homo sapiens]	8gy5	cemiplimab
Monkey [cynomolgus monkey]	AF3 (Q4R367)	
Mouse [mus musculus]	3bik	
Rat [rattus norvegicus]	AF3 (A6JR44)	
Alpaca [lama pacos]	AF3 (A0A6J0ASH2)	
**Transferrin receptor 1 species orthologs**		
Human [homo sapiens]	AF3 (P02786)	OKT9 Fab (as modelled in[1])
Monkey [cynomolgus monkey]	AF3 (A0A2K5X958)	
Mouse [mus musculus]	AF3 (Q62351)	
Rat [rattus norvegicus]	AF3 (Q99376)	
Alpaca [lama pacos]	AF3 (A0A6J0ACT4)	
**Negative controls**		
GLP-1R	6x18	
GFP	5b61	
RNase A	7rsa	
HSA	1ao6	

Table S5: Overview of protein targets, structural models, and binding complexes used for analysis and benchmarking. The table lists the protein systems investigated in this study, including negative control proteins. For each protein or variant, the corresponding molecular structures are given as either experimentally determined structures (PDB IDs) or AlphaFold-predicted structural models (AF3, with UniProt accession numbers in parentheses). Where available, used structures of the protein in complex with a known binder – such as an antibody, ligand, peptide, or Fab fragment – are listed, along with the identity of the interacting molecule. These complexes were used for annotation of binding epitopes, which were exploited for validating surface selectivity, similarity, and discriminability scores. Species orthologs and viral strain variants are indicated where relevant. Negative controls represent structurally unrelated proteins without known binding relevance to the systems under investigation. The used query structure/model is underlined.

References

1. Ferrero, S. *et al.* Antibody-Based Inhibition of Pathogenic New World Hemorrhagic Fever Mammarenaviruses by Steric Occlusion of the Human Transferrin Receptor 1 Apical Domain. *J. Virol.* **95**, e01868-20 (2021).
